# Efficient Preparation of Streptochlorin from Marine *Streptomyces* sp. SYYLWHS-1-4 by Combination of Response Surface Methodology and High-Speed Counter-Current Chromatography

**DOI:** 10.3390/molecules21060693

**Published:** 2016-05-27

**Authors:** Lin Li, Shan He, Lijian Ding, Ye Yuan, Peng Zhu, Slava Epstein, Jianzhong Fan, Xiaokai Wu, Xiaojun Yan

**Affiliations:** 1 LiDakSum Marine Biopharmaceutical Research Center, Ningbo University, Ningbo 315211, China; lilin0574@163.com (L.L.); huahua20062008@126.com (L.D.); 23yuanye@163.com (Y.Y.); 2 Key Laboratory of Applied Marine Biotechnology of Ministry of Education, Ningbo University, Ningbo 315211, China; zhupeng@nbu.edu.cn (P.Z.); wuxiaokai@nbu.edu.cn (X.W.); 3 Department of Biology, Northeastern University, Boston, MA 02115, USA; s.epstein@neu.edu; 4 Ningbo Boao Bioengineering Corporation, Ningbo 315201, China; fan@zjboao.cn

**Keywords:** streptochlorin, *Streptomyces* sp. SYYLWHS-1-4, response surface methodology, high-speed counter-current chromatography, preparation

## Abstract

Since first isolated from the lipophilic extract of *Streptomyces* sp. SF2583, streptochlorin, has attracted a lot of attention because of its various pharmacological properties, such as antibiotic, antiallergic, antitumor, and anti-inflammatory activities. For the efficient preparation of streptochlorin from a producing strain *Streptomyces* sp. SYYLWHS-1-4, we developed a combinative method by using response surface methodology (RSM) and high-speed counter-current chromatography (HSCCC). In the fermentation process, we used RSM to optimize the condition for the efficient accumulation of streptochlorin, and the optimal parameters were: yeast extract 1.889 g/L, soluble starch 8.636 g/L, K_2_HPO_4_ 0.359 g/L, CaCl_2_ 2.5 g/L, MgSO_4_ 0.625 g/L, marine salt 25 g/L, medium volume 50%, initial pH value 7.0, temperature 27.5 °C, which enhanced streptochlorin yield by 17.7-fold. During the purification process, the preparative HSCCC separation was performed using a petroleum ether–ethyl acetate–methanol–water (9:0.8:5:5, *v*/*v*/*v*/*v*) biphasic solvent system, where 300 mg of crude sample yielded 16.5 mg streptochlorin with over 95% purity as determined by UPLC. Consequently, the combination method provided a feasible strategy for highly effective preparation of streptochlorin, which ensured the supply of large amounts of streptochlorin for *in vivo* pharmacological assessments or other requirements.

## 1. Introduction

**Figure 1:**
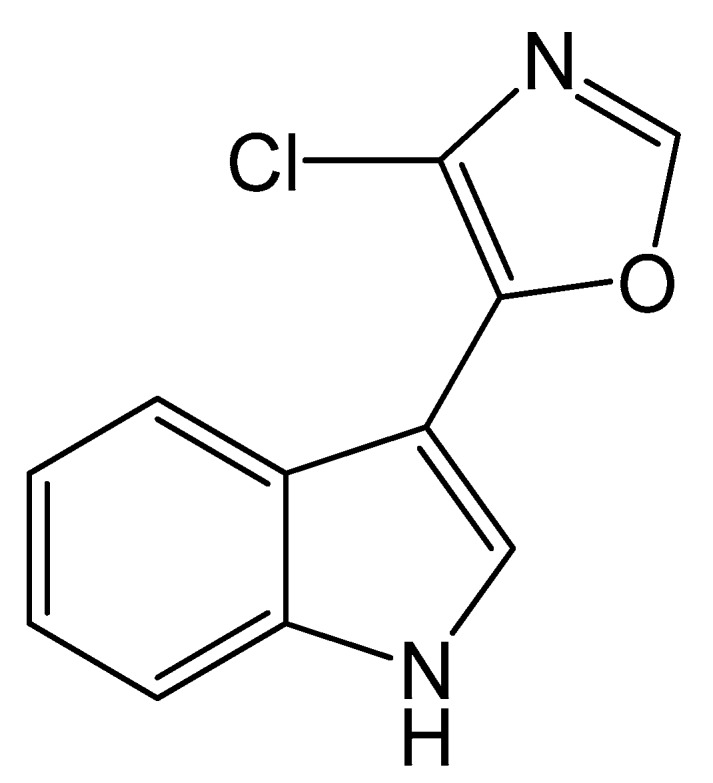
The chemical structure of streptochlorin.

Microbial natural products represent an important source of novel therapeutic agents [1,2]. Streptochlorin (Figure 1), originally isolated from *Streptomyces* strain SF2583 [3], is an indole alkaloid with a variety of significant biological activities, such as antibiotic [4,5], anti-inflammatory [6], antiallergic [7] and antitumor [7,8,9] effects. A marine *Streptomyces* strain SYYLWHS-1-4, which was cultivated from a mangrove sediment sample by microbial trap [10], was found to produce streptochlorin. However, under standard fermentation conditions, the yield of streptochlorin in the spent medium is relatively low, and its isolation and preparation requires complex chromatographic steps, often leading to serious losses. Therefore, optimization of fermentation conditions for streptochlorin production and developing an efficient preparation method are extremely urgent.

Efficient preparation of microbial natural products involves two major steps. Fermentation conditions should be optimized to enhance fermentation yield of target compound(s), followed by development of an efficient separation and purification process. On the one hand, response surface methodology (RSM) is commonly used in building models and optimizing processes. In our previous reports, we successfully applied RSM to optimize fermentation medium components and environmental conditions for different bioactive microbial natural products [11,12]. On the other hand, the conventional isolation chromatographic method for natural products generally contains several steps, which needs plenty of time and results in a serious loss of samples because of irreversible adsorption [13]. High-speed counter-current chromatography (HSCCC), as a unique chromatography without solid support first invented by Ito [14], has an inherent capability to separate components from crude samples with higher efficiency and recovery. It has been widely employed in the isolating of active products from plants and microorganisms [15,16,17,18,19,20].

In this study, we aimed to establish an efficient preparation method for streptochlorin by a combination of RSM and HSCCC. We propose that this combination could be a useful general strategy for microbial natural products.

## 2. Results

### 2.1. Optimization of Culture Conditions by RSM

#### 2.1.1. Determination of the Best Time in Flask Fermentation

**Figure 2:**
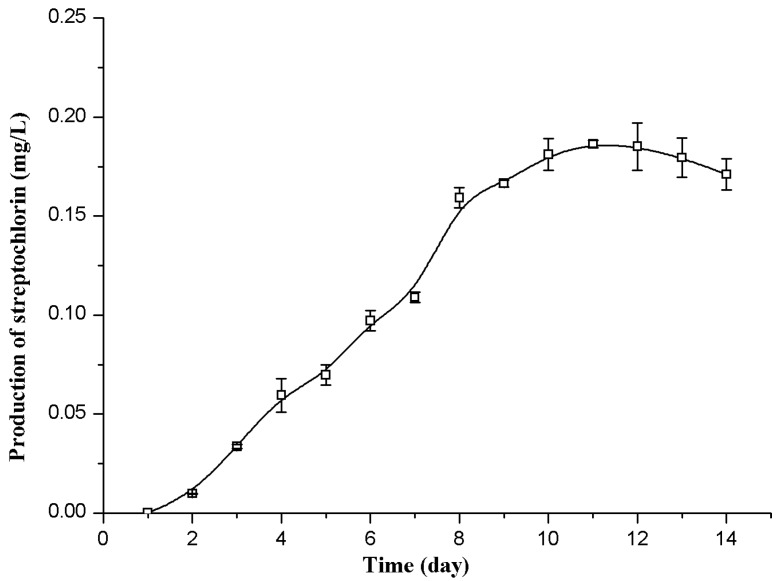
Time courses of streptochlorin production by *Streptomyces* strain SYYLWHS-1-4 in the flask culture.

As shown in Figure 2, the streptochlorin production in the flask fermentation reached the maximal value of 0.18 mg/L on the eleventh day. By employing this fermentation time (11 days), the production of streptochlorin can then be improved using Plackett-Burman design and Box-Behnken design.

#### 2.1.2. Plackett-Burman Design and Identification of Significant Variables that Affect Streptochlorin Production

Plackett-Burman design (PBD) is a powerful tool to identify the most significant factors from lots of variables, minimizing the number of tests. In this study, a total of 20 tests were conducted in triplicate to select the significant variables for streptochlorin production. These include yeast extract, soluble starch, MgSO_4_, K_2_HPO_4_, CaCl_2_, initial pH value, medium volume, temperature and marine salt. Their effect on the response and the level of production are presented in Table 1.

In accordance with the data analyzed by the Minitab software 16 (Minitab Inc., State College, PA, USA), yeast extract (X_1_), soluble starch (X_2_) and K_2_HPO_4_ (X_4_) had confidence levels (*p* < 0.05) and hence influenced the production of streptochlorin markedly. The others (*p* > 0.05) were deemed insignificant. In addition, the *R*^2^_adj_ (0.9445) was close to the *R*^2^ (0.9780) value, which illustrated that the model was adequate. PBD results also indicated that yeast extract, soluble starch and K_2_HPO_4_ exhibited a negative effect on streptochlorin production. According to these results, the three variables, yeast extract (X_1_), soluble starch (X_2_) and K_2_HPO_4_ (X_4_), were selected for further optimization.

#### 2.1.3. Optimization by Box-Behnken Design

To determine the optimal conditions of the three selected factors, RSM using the Box-Behnken design (BBD) was adopted. In this design, the respective levels for the three variables are shown in Table 2, and the concentrations of the other variables were set at zero levels. Data were analyzed by multiple regression analysis methods and an equation model for streptochlorin production was developed as follows:

*Y* = 2.9040 + 0.7703 × X_1_ + 0.5307 × X_2_ + 0.4665 × X_4_ - 0.4688 × X_12_ − 0.5895 × X_22_ − 0.1994 × X_42_ + 0.1315 × X_1_ × X_2_ − 0.2668 × X_1_ × X_4_ − 0.2163 × X_2_ × X_4_ (1)

where *Y* is the predicted streptochlorin production, and X_1_, X_2_ and X_4_ are the coded values of yeast extract, soluble starch and K_2_HPO_4_, respectively. *F*-test was employed to determine its statistical significance. The analysis of variance (ANOVA) for response surface model is presented in Table 3. The high *F*-value (49.54) and a very low *p*-value (<0.001) suggested that the model was highly significant, and the *p*-value (0.12) of “lack-of-fit” indicated that it was not significantly relevant to the pure error. The present *R*^2^ value indicates that the model could explain 98.89% of the response variability, and the *R*^2^_adj_ value of 0.9689 is also able to validate the significance of this model. Therefore, the experimental results and the theoretical values predicted by the polynomial model showed a close agreement, and this model could be used for future study.

The regression coefficients of the model equation and the corresponding *p*-values are listed in Table 4. The *p*-values less than 0.05 indicated that the corresponding coefficient is significant. Table 4 suggests that coefficients of yeast extract (X_1_), soluble starch (X_2_) and K_2_HPO_4_ (X_4_) are highly significant and none of the mutual interactions are significant in streptochlorin production.

**Figure 3:**
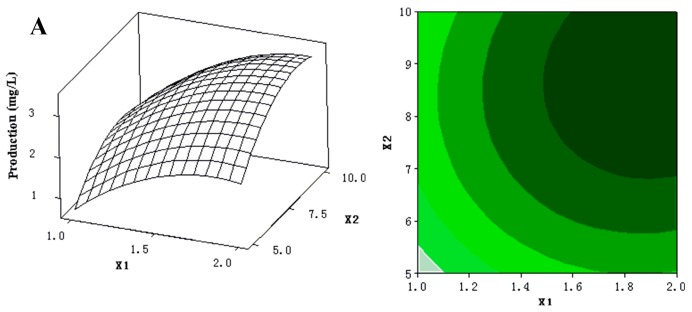

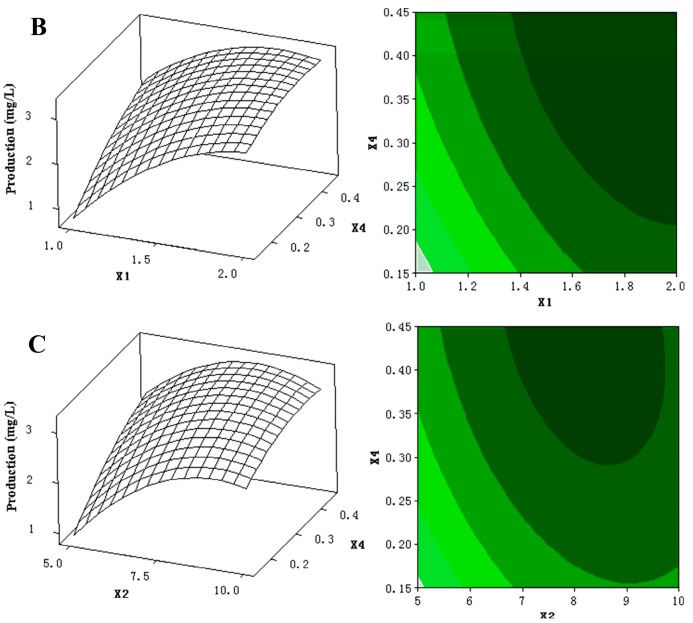
(**A**) 3D response surface curve (**left**) for production (streptochlorin) affected by yeast extract (X_1_) and soluble starch (X_2_), when K_2_HPO_4_ (X_4_) was maintained at 0.3 g/L. Two-dimensional contour plot (**right**) showing production (streptochlorin) in response to varying of yeast extract (X_1_) and soluble starch (X_2_); (**B**) 3D response surface curve (**left**) for production (streptochlorin) affected by yeast extract (X_1_) and K_2_HPO_4_ (X_4_), when soluble starch (X_2_) was maintained at 7.5 g/L. Two-dimensional contour plot (**right**) showing production (streptochlorin) in response to varying of yeast extract (X_1_) and K_2_HPO_4_ (X_4_); (**C**) 3D response surface curve (**left**) for production (streptochlorin) affected by soluble starch (X_2_) and K_2_HPO_4_ (X_4_), when yeast extract (X_1_) was maintained at 1.5 g/L. Two-dimensional contour plot (**right**) showing production (streptochlorin) in response to varying of soluble starch (X_2_) and K_2_HPO_4_ (X_4_).

The 3D response surfaces and 2D contour plots were plotted to provide a visual pairwise combination of the three factors for the streptochlorin production (Figure 3). Furthermore, these graphs can facilitate the determination of the optimal experimental conditions for maximum streptochlorin yield.

#### 2.1.4. Validation of the Optimized Condition

We validated and confirmed the suitability of the equation by further verification experimentation. The fermentation medium components were altered to yeast extract 1.889 g/L, soluble starch 8.636 g/L, K_2_HPO_4_ 0.359 g/L, CaCl_2_ 2.5 g/L, MgSO_4_ 0.625 g/L, marine salt 25 g/L, temperature 27.5 °C, initial pH value 7.0, and medium volume 500 mL. We found that, after 11 days, the value of observed response (3.37 mg/L) essentially matched the maximum predicted yield (3.4179 mg/L), which enhanced streptochlorin yield by 17.7-fold. In addition, the production of streptochlorin was further investigated by a 5 L jar fermenter, which was 4% higher than the flask fermentation. Therefore, we concluded that the model developed is reliable and accurate for predicting the production of streptochlorin.

### 2.2. Preparation of Streptochlorin by HSCCC

#### 2.2.1. UPLC Analysis of the Crude Sample

**Figure 4:**
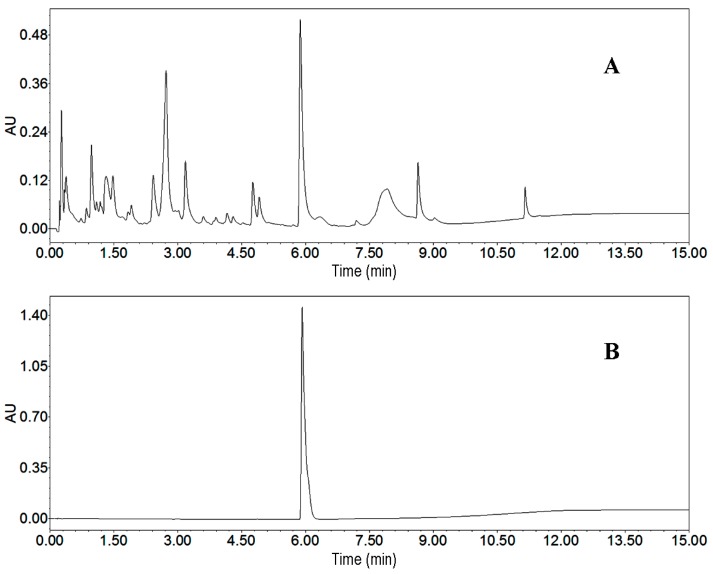
UPLC chromatograms. (**A**) crude sample from *Streptomyces* strain SYYLWHS-1-4; (**B**) HSCCC fraction of peak 2. Conditions: column, Waters ACQUITY UPLC Column (Milford, CT, USA), 100 mm × 2.1 mm, ID, 1.7 μm; column temperature, 25 °C; mobile phase, methanol and water at the gradient (methanol: 0–10 min, 10%–90%; 10–15 min, 90%); flow rate, 0.5 mL/min; detection wavelength, 220 nm.

As the first step, we analyzed the crude sample by UPLC. We employed different elution modes, flow rates and other conditions to develop a best UPLC method. The target compound and impurities were separated markedly when the elution mode was as follows: methanol: 0–10 min, 10%–90%; and 10–15 min, 95%. The flow rate, detection wavelength and column temperature were adjusted to 0.5 mL/min, 220 nm and 25 °C, respectively. The UPLC chromatogram of the crude sample is presented in Figure 4A. According to UPLC peak area percentage, the content of the target compound was 6.5%.

#### 2.2.2. Selection of Suitable Two-Phase Solvent System

In an HSCCC separation, the most critical step is to select an appropriate solvent system. The solvent system should be in accordance with the partition coefficients (*K*_D_) of the target component for HSCCC, 0.5 ≤ K ≤ 2 [14]. A lower *K*_D_ value elutes with lower resolution and a larger *K*_D_ results in broader peaks because of the prolonged elution time. Following the golden rules proposed by Ito [14], we conducted preliminary experiments to select the solvent system of the target compound, and the *K*_D_ values at different volume ratios of the two-phase solvent system (petroleum ether–ethyl acetate–methanol–water) are listed in Table 5. Accordingly, the two-phase solvent system, petroleum ether–ethyl acetate–methanol–water at a volume ratio of 9:0.8:5:5 (*K*_D_ value 1.05) is found to be suitable for the following HSCCC separation.

#### 2.2.3. HSCCC Separation

**Figure 5:**
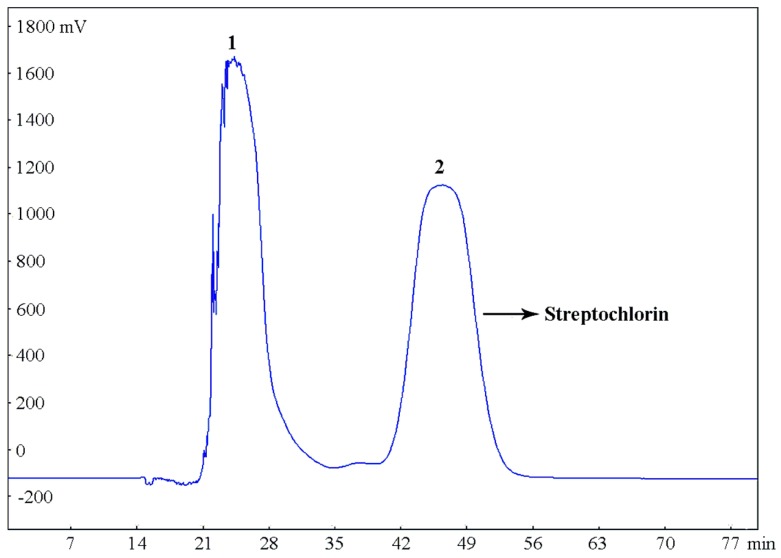
HSCCC chromatogram of the crude extract of *Streptomyces* strain SYYLWHS-1-4 in the solvent system of petroleum ether–ethyl acetate–methanol–water (9:0.8:5:5, *v*/*v*/*v*/*v*), stationary phase: upper phase, mobile phase: lower phase, flow rate: 5.0 mL/min, rotary speed: 800 rpm, column temperature: 25 °C, sample size: 300 mg crude extract dissolved in a 20 mL mixture of upper and lower phases (1:1, *v*/*v*) of solvent system, detection wavelength at 220 nm.

Under optimized conditions, the crude sample (300 mg) was completely dissolved in the selected two-phase solvent system (20 mL) for the separation by HSCCC with the following parameters: rotary speed, 800 rpm (as recommended by the manufacturer); two-phase solvent system, petroleum ether–ethyl acetate–methanol–water (9:0.8:5:5, *v*/*v*/*v*/*v*); flow rate, 5.0 mL/min; column temperature, 25 °C, detection wavelength, 220 nm. The HSCCC separation chromatogram is shown in Figure 5, peak fractions were obtained within 1 h, and the peak 2 fraction eluted from 42 to 52 min in the chromatogram contained the target compound (streptochlorin).

#### 2.2.4. UPLC Analysis and Identification of Peak Fraction

The peak 2 fraction separated by HSCCC was analyzed by UPLC in the same gradient mode as for the crude sample. This chromatogram is given in Figure 4B. The peak fraction collected from the crude sample (300 mg) was evaporated under vacuum to yield 16.5 mg of the target compound, with purities over 95%. Identification of peak 2 fraction was carried out by ESI-MS, ^1^H- and ^13^C-NMR, the detailed data are as follows: High Resolution Electrospray Ionization Mass Spectroscopy (HRESIMS) data: *m*/*z* 219.0303 [M + H]^+^. ^1^H-NMR (500 MHz, CD_3_OD, Tetramethylsilane (TMS)) ppm: 7.28 (2H, dt, *J* = 24.0, 7.4 Hz), 7.45 (1H, d, *J* = 8.1 Hz), 7.81 (1H, d, *J* = 2.7 Hz), 7.88 (1H, s), 8.09 (1H, d, *J* = 7.9 Hz), 8.65 (1H, s). ^13^C-NMR (125 MHz, CD_3_OD, TMS) ppm: 103.93, 111.61, 120.93, 121.32, 121.83, 123.43, 123.46, 124.56, 135.80, 143.34, and 147.72. Furthermore, the structural identification was further proved by two-dimensional NMR. Compared with the data given in the literature [9], it was identified as streptochlorin.

#### 2.2.5. Comparison of Two Chromatographic Systems for Preparative Separation of Streptochlorin

**Figure 6:**
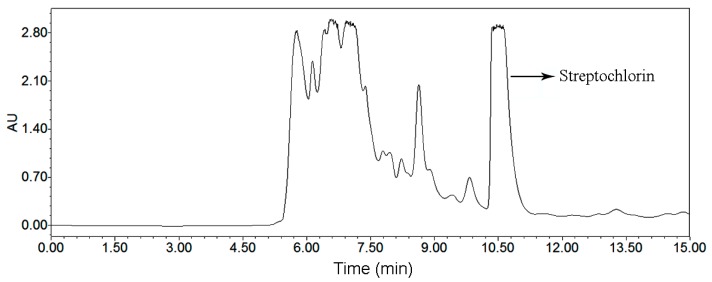
Preparative HPLC chromatogram of the crude extract of *Streptomyces* strain SYYLWHS-1-4, flow rate: 9.0 mL/min, methanol and water at the isocratic mode (65% methanol, 15 min), sample size: 500 μL containing 40 mg crude extract, detection at 220 nm.

The HSCCC and preparative HPLC chromatograms for preparative isolation of streptochlorin are given in Figure 4 and Figure 6, respectively. The comparative study about these two chromatograms is given in Table 6, and the advantages and disadvantages about elution system, sample capacity, organic solvent consumption, productivity, purity and sample recovery of these two chromatographic systems were assessed. HSCCC benefited from obvious advantages when compared with preparative HPLC: sample capacity, productivity and recovery of HSCCC were absolutely high, while solvent consumption and run time were low. In summary, we conclude that the HSCCC method is rapid, economical and efficient in comparison with preparative HPLC method.

## 3. Discussion

Natural product discovery continues in a process of evolution and plays an important role in the drug discovery and development process [1,21,22]. In recent years, research interest has been shifted from terrestrial-derived natural products to marine natural products, because marine organisms have been shown to produce a variety of novel natural products with diverse biological activity, potentially leading to a new wave of drug discovery [23,24]. In addition, about 30% of marine natural products were obtained from microbes [11,21]. Thus, it is critical that marine microbes be pursued as sources of novel natural products.

However, the yields of target microbial compounds under standard fermentation conditions are usually unsatisfactory. As an example, the yield of streptochlorin in standard culture is typically fairly low. By PBD experimentation, we determined that yeast extract (X_1_), soluble starch (X_2_) and K_2_HPO_4_ (X_4_) concentrations were significant factors influencing the production of streptochlorin. The optimized levels of these three factors were investigated by BBD experimentation. The maximum production of streptochlorin predicted by the quadratic model was 3.4179 mg/L. Validation experiments confirmed that the average concentration of streptochlorin was 3.37 mg/L under the optimized conditions, suggesting that the experimental value and predicted value were in good agreement. This represents a 17.7-fold enhancement compared with the standard culture conditions. Furthermore, the validation experiment was performed in a 5 L jar fermenter and the production (3.51 mg/L) of streptochlorin was higher than the productivity of the optimized fermentation in flask. It provided a theoretical foundation for further study on industrial fermentation for streptochlorin production.

In recent years, HSCCC has been successfully applied in the preparative separation of many kinds of natural products because this technique is (a) easy to scale-up, (b) often leads to a maximum sample recovery, (c) allows for an array of solvent choices, which, in turn, allows for considerable flexibility, and (d) is capable of separating ubiquitous materials [14,25]. Nevertheless, HSCCC used in the preparative separation of marine natural products is in its infancy. In our laboratory, an antitumor ubiquinone, three sulfur-containing Diketopiperazines (DKPs) and two macrolactin antibiotics were successfully separated by HSCCC from fermentation products of the marine strain *Pseudoalteromonas rubra*, *Cladosporium* sp. and *Bacillus amyloliquefaciens*, respectively [11,26,27]. In this study, a HSCCC method with a solvent system composed of petroleum ether–ethyl acetate–methanol–water (9:0.8:5:5, *v*/*v*/*v*/*v*) was implemented. In addition, 16.5 mg of streptochlorin was obtained from 300 mg of the *Streptomyces* strain SYYLWHS-1-4 crude extract with ideal purity (˃95%) and high recovery (91%). Compared with preparative HPLC, HSCCC demonstrated its advantages with higher recovery and productivity, illustrating that HSCCC is a rapid, economical and efficient preparative chromatographic method. Nevertheless, it is worth noting that organic synthesis may be an alternative approach for the preparation of this bioactive compound.

## 4. Materials and Methods 

### 4.1. Microorganism and Fermentation Conditions

The *Streptomyces* strain SYYLWHS-1-4 (CGMCC, No. 11468) used in this study was cultivated from a mangrove sediment sample collected in Sanya, Hainan, China, by microbial trap, an *in situ* cultivation device for filamentous actinomycetes [10]. *Streptomyces* sp. SYYLWHS-1-4 was directly inoculated into a 500 mL-Erlenmeyer flask containing 100 mL of the seed medium (soluble starch 20 g/L, MgSO_4_·7H_2_O 0.5 g/L, K_2_HPO_4_ 0.5 g/L, KNO_3_ 1 g/L, FeSO_4_·7H_2_O 0.01 g/L, marine salt 17.5 g/L. pH value 7.4–7.6). The flasks were cultured at 28 °C with shaking at 150 rpm. After 2 days of cultivation, 5% (*v*/*v*) seed cultures were transferred into a 1000 mL-Erlenmeyer flask containing 500 mL fermentation medium. The flasks were incubated on a rotary shaker at 150 rpm for 14 days, with samples taken periodically to determine the time course of streptochlorin production. All the chemicals were purchased from Sinopharm Chemical Reagent Co. Ltd. (Shanghai, China).

### 4.2. Experimental Design and RSM Optimization

In the present study, PBD was used to determine the most influential of the nine variables for streptochlorin yield, and the variables with a confidence level (*p* < 0.05) were regarded as significant factors [28]. The PBD experimental design is given in Table 1. The following step was to determine the optimal conditions of the three most significant factors. For this purpose, BBD was applied. In this study, the BBD consisted of 15 trials, each conducted in triplicate. The experimental data were analyzed with the Minitab16 software (Minitab Inc., State College, PA, USA) to fit the second-order polynomial model equation:

*Y* = β_0_ + β_1_ × X_1_ + β_2_ × X_2_ + β_4_ × X_4_+ β_11_ × X_12_ + β_22_ × X_22_ + β_44_ × X_42_ + β_12_ × X_1_ × X_2_ + β_14_ × X_1_ × X_4_ + β_24_ × X_2_ × X_4_ (2)

where *Y* is the predicted response, and X_1_, X_2_ and X_4_ are the coded variables. β_0_ is the intercept. The regression coefficients (β_1_, β_2_ and β_4_) are linear coefficients. β_11_, β_22_ and β_44_ represent quadratic coefficients. β_12_, β_14_ and β_24_ refer to interactive coefficients. Minitab16 was also used to perform ANOVA. After determining the optimum fermentation medium, *Streptomyces* strain SYYLWHS-1-4 was fermented in our optimized medium for validation.

### 4.3. Production of Streptochlorin from the Streptomyces Strain SYYLWHS-1-4 by a Jar Fermenter

Spores of *Streptomyces* sp. SYYLWHS-1-4 were spread into a 500 mL-Erlenmeyer flask with 150 mL of media composed of: yeast extract (1.889 g/L), K_2_HPO_4_ (0.359 g/L), soluble starch (8.636 g/L), CaCl_2_ (2.5 g/L), MgSO_4_ (0.625 g/L), marine salt (25 g/L), pH value 7.0. The flasks were incubated at 27.5 °C with shaking at 150 rpm for 48 h to yield the seed liquid. Then, the seed liquid was transferred to a 5 L-fermenter (Shanghai Guoqiang Bioengineering Equipment Co., Ltd., Shanghai, China) containing 3.5 L of the same liquid medium and incubated with agitation at 300 rpm and dissolved oxygen (40%) at 27.5 °C. After 11 days, the concentration of streptochlorin in the fermentation broth was analyzed by UPLC.

### 4.4. UPLC Analysis, Preparative HPLC Separation and Structure Identification

UPLC analysis were all carried out on a Waters ACQUITY UPLC system, including a quaternary solvent deliver system, an ACQUITY UPLC Photodiode Array (PDA) eλ Detector (Waters Co., Milford, CT, USA), and an autosampler (Waters Co., Milford, CT, USA). A reverse-phase ACQUITY UPLC column (100 mm × 2.1 mm, ID, 1.7 μm; Waters Co., Milford, CT, USA) was applied for the UPLC analyses. We used methanol-water (methanol: 0–10 min, 10%–90%) as the mobile phase. The effluent was continuously monitored at 220 nm and the flow rate was 0.5 mL/min. The preparative HPLC separation was performed using an YMC-Pack ODS-A column (250 mm × 20 mm ID, 5 μm; YMC, Kyoto, Japan). A Waters 600 system (Waters) was carried out for the preparative HPLC separation of streptochlorin. The mobile phase was methanol-water (65%:35%) at a flow rate of 9 mL/min, and the detection wavelength was 220 nm. The chemical structures of the peak fractions were identified by ESI-MS, ^1^H-NMR, and ^13^C-NMR analysis and compared with the published literatures.

### 4.5. Preparation of Crude Sample

The *Streptomyces* strain SYYLWHS-1-4 was cultivated at the optimized conditions (yeast extract 1.889 g/L, soluble starch 8.636 g/L, K_2_HPO_4_ 0.359 g/L, CaCl_2_ 2.5 g/L, MgSO_4_ 0.625 g/L, marine salt 25 g/L, medium volume 500 mL, initial pH value 7.0, and culture temperature 27.5 °C) in 1000 mL-Erlenmeyer flasks with shaking at 150 rpm for 11 days. The whole fermentation broth (25 L) of the bacterium was extracted with ethyl acetate (3 × 25 L). All the ethyl acetate extracts were then concentrated under reduced pressure at 37 °C. Finally, 1.3 g of crude sample was obtained and prepared for the further separation.

### 4.6. Evaluation of Partition Coefficient Value

*K*_D_ values of the given compound were determined by UPLC as follows: 3 mL of the two-phase solvent system (petroleum ether–ethyl acetate–methanol–water) was mixed in an Eppendorf tube, a suitable crude sample (3 mg) was weighed into the tube immediately. The Eppendorf tube was dynamically shaken for 3 min to dissolve the crude sample. Then, the mixture solvent was divided into two layers by centrifugation (3000 rpm, 3 min). Equal volumes of each phase were taken out and analyzed by UPLC. The partition coefficient values are expressed by dividing the peak area of the target compound in the upper phase by that in the lower phase.

### 4.7. Preparation of Solvent System and Sample Solution for HSCCC Separation

In the present paper, the two-phase solvent system consisting of petroleum ether–ethyl acetate–methanol–water (9:0.8:5:5, *v*/*v*/*v*/*v*) was determined according to the *K*_D_ values of the target compound. The solvent system was mixed in the separation funnel by vigorously shaking in accordance with the selected volume ratio. Then, the two phases were separated at room temperature. Both upper and lower phases were degassed by ultrasonic bath for 15 min prior to use. In addition, the crude sample (300 mg) was dissolved in the determined two-phase solvent system (20 mL), which was prepared as the sample solution for the following separation by HSCCC.

### 4.8. HSCCC Separation

The preparative HSCCC apparatus applied in the present study was a TBE-300C high-speed counter-current chromatograph (Tauto Biotech Co., Ltd., Shanghai, China). Separation was carried out in “head-to-tail” elution mode. Initially, the coiled column (320 mL) was entirely filled with the upper phase as the stationary phase. Then, the HSCCC instrument was rotated at a revolution speed of 800 rpm. Meanwhile, the lower phase (mobile phase) was pumped through the column at a flow rate of 5 mL/min. When the lower phase was flowed out, the sample solution was injected into the column through the injection valve. The separation temperature was controlled at 25 °C, and the effluent was monitored by a UV detector (Shanghai Sanotac Scientific Instruments Co. Ltd., Shanghai, China) at 220 nm. According to the HSCCC elution chromatogram, peak fractions were collected manually.

## 5. Conclusions

The combination of RSM and HSCCC as a powerful strategy was successfully applied for efficient preparation of streptochlorin from marine *Streptomyces* sp. SYYLWHS-1-4. The combination method may solve the sample supply problem for at least some of the microbial natural products. We proposed that this combination of RSM and HSCCC could be an efficient protocol for drug discovery from marine microbes.

## Figures and Tables

**Table 1:** The Plackett-Burman design for screening variables in streptochlorin production.

Variable	Code	Low Level	High Level	Coefficient	*t-*Value	*p*-Value
Intercept				1.0930	17.44	0.000
Yeast extract (g/L)	X_1_	2	3	−0.3460	−5.52	0.000
soluble starch (g/L)	X_2_	10	15	−0.1520	−2.42	0.036
MgSO_4_ (g/L)	X_3_	0.5	0.75	−0.0950	−1.52	0.161
K_2_HPO_4_ (g/L)	X_4_	0.5	0.75	−0.1980	−3.16	0.010
CaCl_2_ (g/L)	X_5_	2	3	0.0370	0.59	0.568
Initial pH value	X_6_	6	8	0.0100	0.16	0.876
Medium volume (%)	X_7_	40	60	−0.0330	−0.53	0.610
Temperature (°C)	X_8_	25	30	−0.0840	−1.34	0.210
Marine salt (g/L)	X_9_	20	30	0.1130	1.80	0.102

**Table 2:** The level of variables for the Box-Behnken design.

Variables	Code	Level		
		−1	0	1
Yeast extract (g/L)	X_1_	1	1.5	2
Soluble starch (g/L)	X_2_	5	7.5	10
K_2_HPO_4_ (g/L)	X_4_	0.15	0.3	0.45

**Table 3:** Analysis of variance (ANOVA) for the second-order polynomial model.

Source	SS	*df*	MS	*F* Value	*p*-Value
Model	11.2888	9	1.25431	49.54	<0.001
Residual	0.1266	5	0.02532		
Lack of Fit	0.1162	3	0.03874	7.48	0.120
Pure Error	0.0104	2	0.00518		
Cor Total	11.4154	14			

SS: sum of squares, *df*: degree of freedom, MS: mean square.

**Table 4:** Regression results of the Box–Behnken design.

Variables	Coefficient	*p*-Value
Intercept	2.9040	<0.001
X_1_	0.7703	<0.001
X_2_	0.5307	<0.001
X_4_	0.4665	<0.001
X_1_^2^	−0.4688	0.002
X_2_^2^	−0.5895	0.001
X_4_^2^	−0.1994	0.006
X_1_ × X_2_	0.1315	0.500
X_1_ × X_4_	−0.2668	0.162
X_2_ × X_4_	−0.2163	0.153

**Table 5:** The distribution constants (*K*_D_) of the target compounds at different ratios of volume in petroleum ether-ethyl acetate-methanol-water.

Petroleum Ether–Ethyl Acetate–Methanol–Water	*K*_D_
5:5:5:5	6.47
8:2:5:5	3.47
9:1:5:5	1.33
9:0.8:5:5	1.05
10:0:5:5	0.12

**Table 6:** Comparison of HSCCC and Preparative HPLC.

	HSCCC	Preparative HPLC
Stationary phase	Upper phase petroleum ether–ethyl acetate–methanol–water (9:0.8:5:5, *v*/*v*/*v*/*v*)	YMC C18 column 250 mm × 20 mm ID 5 μm
Mobile phase	Lower phase	Methanol–water (65:35, *v*/*v*)
Sample capacity per run (mg)	300	40
Run time (min)	55	20
Organic solvent consumption (L/mg)	0.048	0.15
Productivity (mg/min)	0.3	0.06
Purity of isolated compound	97%	98%
Sample recovery	91%	48%

## Abbreviations

The following abbreviations are used in this manuscript:

RSM: Response surface methodology
HSCCC: High-speed counter-current chromatography
PBD: Plackett-Burman design
BBD: Box-Bohnken design
UPLC: Ultra Performance Liquid Chromatography
HPLC: High Performance Liquid Chromatography
ESI-MS: Electro Spray Ionization-Mass Spectrometry
NMR: Nuclear Magnetic Resonance
HRESIMS: High Resolution Electrospray Ionization Mass Spectroscopy
TMS: Tetramethylsilane
DKPs: Diketopiperazines
